# Application of bioinformatics-coupled experimental analysis reveals a new transport-competent nuclear localization signal in the nucleoprotein of Influenza A virus strain

**DOI:** 10.1186/1471-2121-9-22

**Published:** 2008-04-28

**Authors:** Krishna Mohan V Ketha, Chintamani D Atreya

**Affiliations:** 1 Section of Cell Biology, Laboratory of Cellular Hematology, Division of Hematology, Office of Blood Research and Review, Center for Biologics Evaluation and Research (F.D.A.) Bethesda, MD 20892, USA

## Abstract

**Background:**

Two nuclear localization sequences (NLS) in influenza A virus nucleoprotein (NP) have been demonstrated to be critical for nuclear import of NP and viral ribonucleoprotein complexes. However, a deletion mutant lacking these two signals was still able to localize to the nucleus suggesting the presence of yet another (a third) potential NLS in the NP protein. In order to identify the nature of this potential NLS signal in the NP of a WS/33L influenza virus A strain, we utilized the tools of bioinformatics coupled with functional experimental analyses in the present study.

**Results:**

Comparison of the deduced aa sequence of NP of WS/33L strain with the published WS/33 NP sequences revealed that a single amino acid (aa) change (Met to Arg) at position 105 results in converting the flanking regions (between aa position 90–121, a 32-residue stretch) into two classical overlapping bipartite NLS (obpNLS). GenBank search revealed that 9 out of 500 published NP sequences contain a similar Arg at position 105 (instead of Met) with a 100% homology to the obpNLS region. Various NP-green fluorescent protein (GFP) fusion constructs with and without the signal (obpNLS-Arg^105^) were utilized to understand the functional nature of this signal. We analyzed the transport competency of the expressed chimeric proteins in terms of their cellular localization by confocal immunofluorescence assay. Our analysis revealed that all NP-GFP constructs containing the wild-type (R^105^) sequence localized predominantly to the nucleus. Constructs lacking the obpNLS or constructs with reverse mutation (R^105 ^to M^105^) on the other hand exhibited predominant cytoplasmic localization pattern. Interestingly, when the 32 aa obpNLS was fused with an unrelated viral protein (rotavirus NSP6) that has been known to be cytoplasmic protein, the chimeric protein (obpNLS-NSP6) was efficiently transported into the nucleus, indicating an efficient nuclear transport function of the 32-residue obpNLS in the NP of WS/33L strain of influenza A virus.

**Conclusion:**

This report while not only establishing a new NLS in the influenza A virus strain, it also reinforces the idea that proper application of bioinformatics-coupled experimental analysis serves as a powerful tool in identifying new functional signals in proteins of interest.

## Background

Influenza A virus is a negative-stranded RNA virus with 8 genomic segments coding for at least 11 proteins [1]. Gene segment 5 encodes the 498-amino acids (aa) long nucleoprotein (NP) that has been shown to be a multifunctional protein with critical roles during various stages of the viral life cycle. The viral RNA tightly bind to NP and polymerase subunit proteins, PB1, PB2 and PA, resulting in the formation of viral ribonucleoproteins (vRNPs) a prerequisite for successful transcription and replication [1-5]. The primary role of NP as an RNA-binding protein and thus its role as a structural protein contributing towards the formation of the RNP complex within the virion is clearly evident. However, it is the series of events following infection that delineates the major function of NP, which mainly constitute importing of the vRNP complex into nucleus, then exporting it back to cytoplasm, and finally preventing their reentry into nucleus [1-3,6-10]. Interaction of NP with several other viral and host proteins is critical for this nuclear import and export [1,11-13].

Nuclear import of NP is regulated by two nuclear localization signals (NLS), a non-conventional NLS (nNLS) and a classical NLS (cNLS) present within the NP [3,8,10,14,15]. A nuclear import signal in a protein is characterized by one (monopartite) or two (bipartite) short stretches of basic amino acids [16-18]. The monopartite signals comprise of two types: 4 residue pattern (called "pat4") or the 7 residue pattern (called "pat7"). Pat4 NLS comprises of either a stretch of 4 basic amino acids (K or R) or 3 basic amino acids (K or R) with the fourth aa being either H or P. Pat7 is a pattern starting with P and followed within 3 residues by a basic segment containing 3 K/R residues out of 4. Thus, based on the above classification the nNLS of NP (^3^**S**x**GTKRSY**xx**M**^13^, important residues highlighted in bold) is aptly termed non-conventional NLS as it does not fall under both pat4 or pat7 type of signals [13,14]. The second NLS of NP (cNLS) on the other hand though, has been classified as a bipartite signal (^198^**KR**GINDRNFWRGENG**RK**T**R**^216^, important residues highlighted in bold) and is located near the middle of the polypeptide [15-18]. Deletion and mutation analysis of both the nNLS and cNLS reveal that these signals are essential for nuclear import, viral mRNA synthesis, vRNA transcription, replication and nucleolar accumulation [3,7,8,10,13-15]. Interestingly, a mutant NP lacking both of these NLSs was still transported to the nucleus suggesting the existence of at least one additional NLS between the cNLS and C-terminus region of NP [8,14].

We report here a third novel overlapping bipartite NLS (obpNLS) in the NP of WS/33L strain located between the nNLS and cNLS regions identified by bioinformatic analyses. Our analysis revealed a single amino acid change (M to R) at position 105 resulted in converting a 32-aa stretch into two obpNLS (^90^**KK**TGGPIYRRVDG**K**W**RR**^106 ^and ^105^**RR**ELILYDKEEI**RR**IW**R**^121^, residues constituting bpNLS are in bold). Using full-length and deletion constructs of NP in fusion with GFP, we present experimental evidence that the obpNLS is indeed a stand alone functional transport signal and supports efficient translocation of the chimeric protein to the nucleus. Furthermore, comparative analysis of 500 NP sequences revealed that 9 influenza virus strains contain NP with similar aa change (R instead of M) at position 105 with 100% homology to the obpNLS region, suggesting the authenticity of the identified amino acid change as a natural variant.

## Methods

### Cells, virus and reagents

MDCK (ATCC, Manassas, VA) and COS-7L cells (Invitrogen, Gaithersberg, MD) were grown at 37°C with 5% CO2 in Dulbecco's modified Eagle medium (DMEM) supplemented with 10% fetal calf serum and penicillin-streptomycin (Invitrogen, Gaithersberg, MD). Influenza A virus WS/33 strain was a gift from Dr. Zhiping Ye, CBER, FDA and is referred to in the present study as WS/33L to distinguish it from the published WS/33 strains. Mouse monoclonal anti-GFP antibody was purchased from Clontech labs (Pasadena, CA).

### RT-PCR

MDCK cells were infected with WS/33L as per a previous protocol and incubated for 48 hours [19]. Total cellular RNA from virus-infected MDCK cells was extracted with RNA STAT-60 as previously described [20]. The RNA (5 μl) was subjected to reverse-transcriptase-based polymerase chain reaction (RT-PCR) using a pair of forward (5'-cgc gaattcatggcgaccaaaggc) and reverse (3'-gacccc gggcccattgtcgtactcctc) primers specific to 5' and 3' orf termini of influenza A nucleoprotein gene [WS/33 strain, GenBank:AAA43452]. PCR products originating from virus-infected cellular RNA were cloned into a TA cloning plasmid, pCR3.1 and referred to as pCR-NP-L. (Invitrogen, Gaithersberg, MD). The PCR products as well as the pCR-NP-L clones were sequenced using internal NP-specific, T7, and BGH primers as provided by the manufacturer (Invitrogen, Gaithersberg, MD).

### Construction of NP-GFP fusion plasmids

Primers were designed based on the Genbank WS/33 NP sequence [GenBank:AAA43452] to generate the full-length and various deletion constructs between aa position 41 and 197 of the NP protein. PCR-NP-L was used as template for PCR reactions and all the originating products were cloned into pEGFP-C2 vector (Clontech, CA) in fusion with the C-terminus of GFP using appropriate restriction enzymes (Table 1). NPΔ obpNLS clone was constructed by inserting the *EcoRI-ApaI *digested fragment of NP^122–197 ^into NP^41–89 ^clone. To derive the obpNLS-NSP6 clone, obpNLS was cloned at the *XhoI-EcoRI *sites of GFP-NSP6 clone such that the N-terminus of obpNLS was in fusion with GFP, whereas the C-terminus of the protein was in fusion with NSP6 protein from lamb rotavirus [21]. All clones were sequenced prior to immunoblot or localization analysis.

**Table 1:** Primers used in the study

S. No.	Clone	Primer No.	Sequence	Restriction sites
1	NP-FL	35104	5'-cgcgaattcatggcgaccaaaggc	*EcoRI*
		35575	3'-gaccccgggcccattgtcgtactcctc	*ApaI*
2	obpNLS+NC	36823	5'-cgc gaattcatccaaatgtgcaccg	*EcoRI*
		62638	3'-cgcgggccctttgatcattctgat	*ApaI*
3	obpNLS+NTer	39815	5'-gggctcgagcatccaaatgtgc	*XhoI*
		39821	3'-gcggaattcgcgccagattcgtct	*EcoRI*
4	obpNLS+CTer	39820	5'-gggctcgagcaagaaaactggagga	*XhoI*
		62638	3'-cgcgggccctttgatcattctgat	*ApaI*
5	bpNLS-1R	39815	5'-gggctcgagcatccaaatgtgc	*XhoI*
		39813	3'-gcgcccgggtcatctcctccactt	*ApaI*
6	bpNLS-1M	39815	5'-gggctcgagcatccaaatgtgc	*XhoI*
		39818	3'-gcgcccgggtcatctcatccactt	*ApaI*
7	bpNLS-2R	39814	5'-gcggaattcaggagagaactcatc	*EcoRI*
		62638	3'-cgcgggccctttgatcattctgat	*ApaI*
8	bpNLS-2M	39819	5'-gcggaattcatgagagaactcatc	*EcoRI*
		62638	3'-cgcgggccctttgatcattctgat	*ApaI*
9	NPΔ obpNLS	39815	5'-gggctcgagcatccaaatgtgc	*XhoI*
		40466	3'-gcggaattcaggatctttccc	*EcoRI*
		39817	5'-gaggaattccaagctaataatggt	*EcoRI*
		62638	3'-cgcgggccctttgatcattctgat	*ApaI*
10	NP^41–89^	39815	5'-gggctcgagcatccaaatgtgc	*XhoI*
		40466	3'-gcggaattcaggatctttccc	*EcoRI*
11	NP^122–197^	39817	5'-gaggaattccaagctaataatggt	*EcoRI*
		62638	3'-cgcgggccctttgatcattctgat	*ApaI*
12	2bpNLS+NSP6	39820	5'-gggctcgagcaagaaaactggagga	*XhoI*
		39821	3'-gcggaattcgcgccagattcgtct	*EcoRI*

### Transfection and confocal microscopic analysis

Transient transfections were performed on COS-7L cells, maintained either in 6-well culture plates or in 8-well chamber slides as described previously [22]. Cells were transfected with 2–5 μg of plasmid DNA with lipofectamine plus^® ^reagent as per the manufacturer's instructions (Invitrogen, USA). Following 24 hours after transfection, cells were fixed in ice-cold acetone and mounted with Vectashield containing propidium iodide (Vector labs, CA). Visualization, analysis and photography were all performed using a Carl Zeiss laser-scanning confocal microscope (Model: LSM5 PASCAL) equipped with a microprocessor. Images were transferred to PC version of Adobe Photoshope 5.0 for labeling and printing.

### Cytolysate preparation and Immunoblot assay

COS-7L cells, cultured in 6–well plates and transfected with various NP-GFP constructs, were washed with 1× PBS, centrifuged at 2.5 K in a refrigerated centrifuge (Sorvall, USA) and extracted with RIPA buffer (50 mM Tris-Cl, pH 8.0, 150 mM NaCl, 0.1% SDS, 1.0% NP-40, 0.5% sodium deoxycholate). Total proteins were resolved on 4–20% SDS-PAGE (Invitrogen, CA), transferred to PVDF membrane (Millipore, USA) and the membrane incubated overnight with 1: 1,000 diluted mouse monoclonal anti-GFP antibody at 4°C [23]. Secondary incubation with 1:10,000 diluted anti-mouse peroxidase-conjugated antibody (Chemicon, USA) was carried out at room temperature for 90 min. and signals were detected by ECL-chemiluminescence detection (Pierce, CA).

## Results and Discussion

### i. Comparative analysis of the deduced amino acid NP sequence from WS/33L

**Figure 1:**
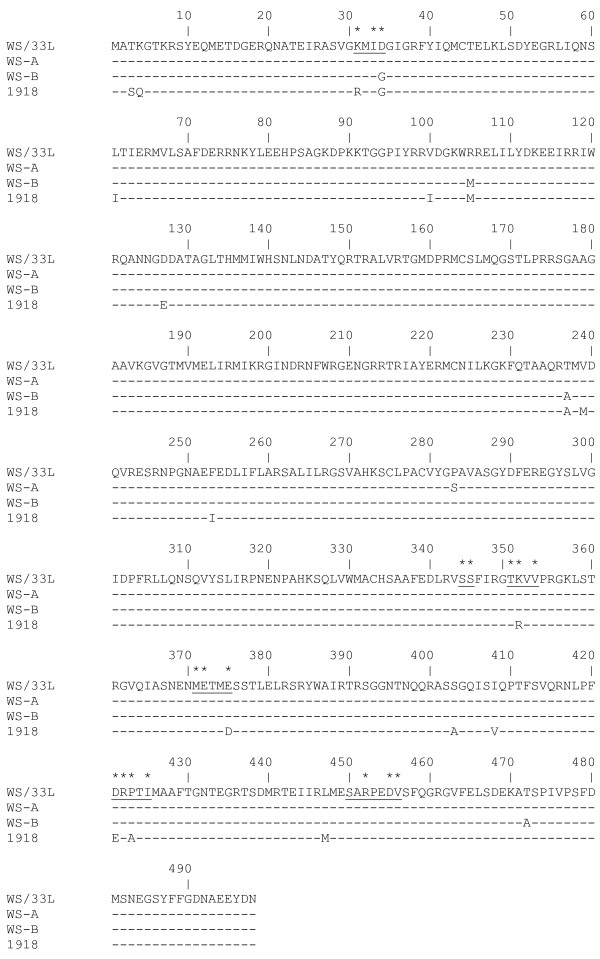
**Amino acid (aa) comparison of WS/33 NP sequences.** Sequence generated from WS/33L in the present study is compared with 5 other WS/33 sequences represented as WS-A [includes sequences EMBL:Q1K9H2, EMBL:AAG41965 and EMBL:ABF21292] and WS-B [includes sequences GenBank:AAA43452 and GenBank:P15682]. NP sequence of the 1918 pandemic flu strain [GenBank: ] is also included for comparison. Identical amino acids are represented by "-"; Phylogenetically important regions (PIR) are underlined; phylogenetically important positions (PIP) are indicated by an asterisk.

The full-length orf of the NP was amplified, cloned, sequenced and the sequence submitted to the genomic database [GenBank: EU330203]. The deduced aa coding sequence of NP-L was compared to five other WS/33 NP sequences available in the database (one of those being only a partial coding sequence between aa 14–193) (Fig. 1). Results from our comparative analyses revealed that the published WS/33 NP sequences fall into two distinct types, type WS-A [consisting of sequences EMBL:Q1K9H2, EMBL:AAG41965 and EMBL:ABF21292] and type WS-B [includes sequence GenBank:AAA43452 and GenBank:P15682] (Fig 1). Between type A and B there is a 98.99% homology at the aa level and differ only by 5 amino acids at residues 34, 105, 237, 283 and 472. NP sequence from the influenza virus strain that caused pandemic flu in 1918 was included here for comparison. Our NP-L sequence appears more like a type A sequence as it shares homology to type A at 4 of the above 5 critical residue positions. However, NP-L sequence shared homology to type B sequences at residue 283, indicating that it does not share a 100% homology to either type A or type B (Fig. 1).

### ii. Bioinformatic analyses of NP-L reveal a novel overlapping bipartite NLS (obpNLS)

**Figure 2:**
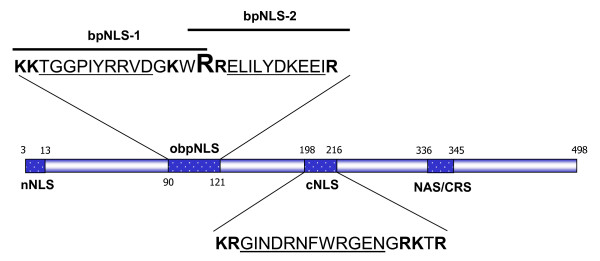
**Schematic representation of various domains of the NP.** Numbers indicate amino acid positions. nNLS: non-conventional nuclear localization signal; cNLS: classical nuclear localization signal; NAS/CRS: nuclear accumulation signal/cytoplasmic retention signal; obpNLS: overlapping bipartite nuclear localization signal; bpNLS: bipartite nuclear localization signal. Critical amino acids contributing to a bpNLS motif are represented by two basic aa-rich domains (in bold) and the spacer 10 aa are underlined. Also note that the cNLS contains a slightly longer (12 aa) spacer region and thereby does not strictly qualify as a classical bpNLS by the PSORT program.

The putative amino acid sequence of NP was analyzed by a web-based PSORT program served at the Institute for Medical Science, University of Tokyo, Japan (see Availability and requirements section for URL). The analyses indicated that a single aa change (M105R) resulted in converting the flanking regions (between aa 90–121) into two overlapping bipartite nuclear localizing signals (obpNLS). The obpNLS constituted of bpNLS-1 (^90^**KK**TGGPIYRRVDG**K**W**RR**^106^) and bpNLS-2 (^105^**RR**ELILYDKEEI**RR**IW**R**^121^) (Fig. 2). It is to be noted that though a second combination, predictive for bpNLS-2, existed between aa 103 and 118 (**K**W**RR**ELILYDKEEI**RR**) we did not utilize this sequence in our localization experiments for the sake of ease of understanding (Fig. 2). Both bpNLS-1 and bpNLS-2 signals adhere strictly to the classical bpNLS signature pattern that consists of 17 aa, with two basic rich regions separated by a spacer region of any 10 aa, and of the two basic regions, one end containing at least two basic aa (K or R) whereas the other flanking end containing at least 3 basic aa out of a total of 5 residues [16-18]. Interestingly, the previously reported classical NLS (cNLS) (between aa 198–216) did not qualify as a classical bpNLS by the PSORT analyses as it had more than 10 aa in the spacer region [15-17]. The above finding is in concurrence with results from a recent study wherein the mechanism by which influenza A virus NP oligomerizes and subsequently binds to RNA were evaluated by structural analysis [5]. In this study, structural analysis of NP was performed in great detail and based on the accessibility of the NLS in relation to the structure, it was suggested that the cNLS may not function as a classical NLS at all [5].

### iii. NP-L full-length and deletion GFP constructs and analysis of their expression

**Figure 3:**
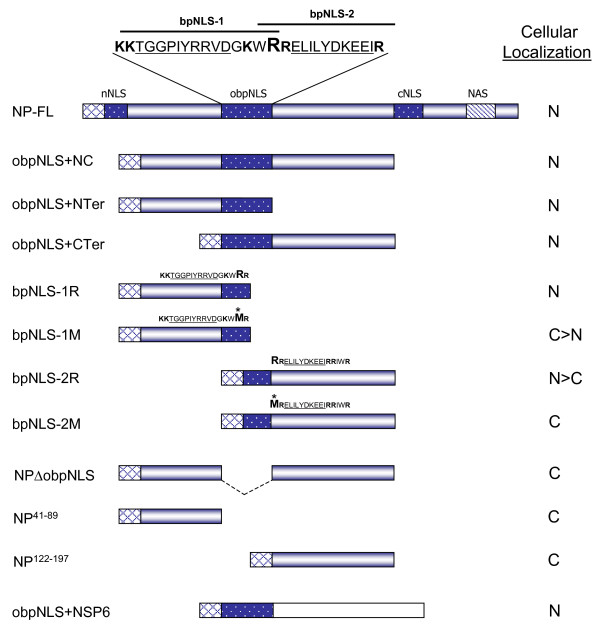
**Schematic representation of NP-full-length and deletion constructs.** Various NP-FL and deletion clones were made between the nNLS and cNLS regions. GFP is represented by chequered-box; NLS regions are indicated by darkly-shaded box; nuclear accumulation signal (NAS) is indicated by striped-box; rotavirus NSP6 is represented by an empty, unshaded box. Critical amino acid (R or M) at position 105 is indicated by larger font size and an asterisk. Cellular localization of each construct is indicated by N (nuclear), N>C (nuclear greater than cytoplasm), C>N (cytoplasm greater than nuclear), and C (cytoplasmic).

**Figure 4:**
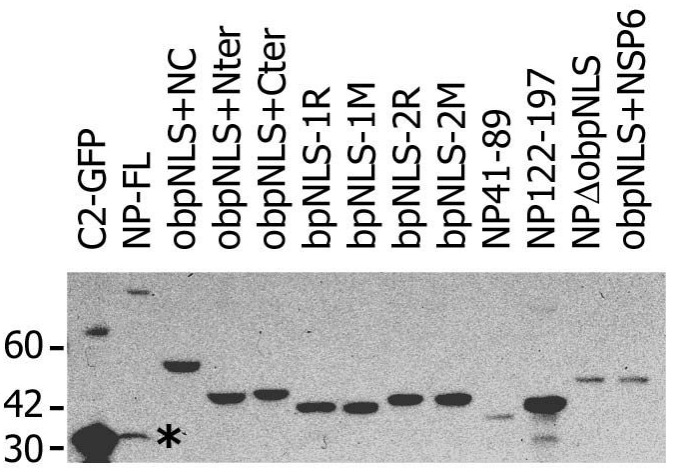
**Immunoblot analysis of NP-GFP constructs. **All NP-GFP plasmid constructs and the empty vector C2-GFP (control) were transfected into COS-7L cells and cell lysates were analyzed to confirm expression of the fusion proteins. Molecular weight markers (in kDa) are indicated on the left. The caspase-cleaved N-terminal fragment of NP-L is indicated by an asterisk (*).

In order to gain a clear understanding of the contribution of the obpNLS in the nuclear localization function of the NP, we designed constructs that excluded nNLS and cNLS regions and contained only the obpNLS and its flanking regions between aa 41–197 (Fig. 3). All NP-GFP plasmid constructs were transfected in to COS-7L cells to analyze the correct (in-frame) expression of the fusion protein. Initially, expression analysis was performed by visually observing for GFP fluorescence under a Nikon Eclipse 2000 fluorescent microscope. Following this, expression of all constructs was confirmed more specifically by analyzing the transfected lysates in an immunoblot assay using a monoclonal GFP antibody. Fig. 4 demonstrates that all the NP-GFP transfected lysates contain fusion proteins within the expected range of size.

### iv. Protein localization analysis by Confocal microscopy

#### a. obpNLS-containing NP-L constructs localize exclusively to the nucleus

**Figure 5:**
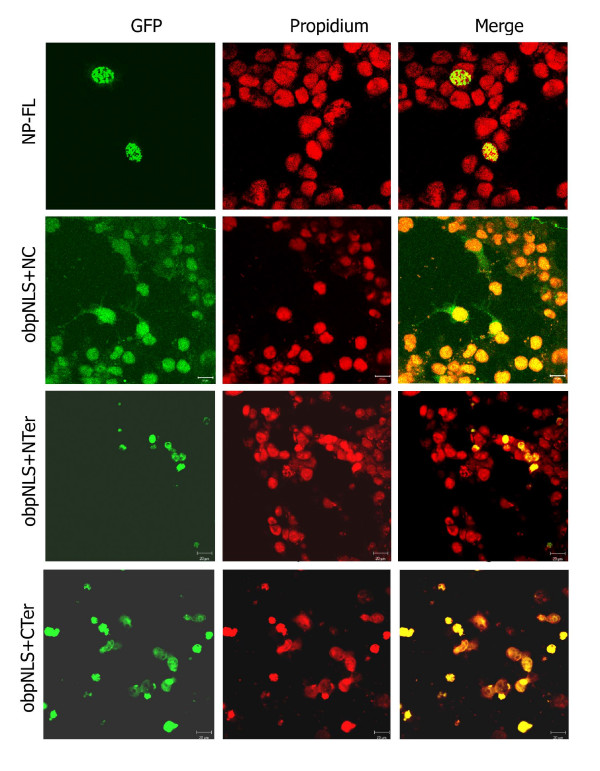
**Confocal immunofluorescence analysis of various NP constructs containing the obpNLS. **COS-7L cells transfected with the various NP-GFP constructs were fixed and mounted with propidium iodide-containing medium to indicate nuclear staining. NP-L exhibits a typical punctuate, nuclear localization pattern. obpNLS+NC, obpNLS+N-ter and obpNLS+C-ter all localize to the nucleus.

Cellular localization of the GFP-fused NP constructs was performed on COS-7L cells and subcellular localization was assessed as described earlier [7]. Localization was classified into N (nuclear), N>C (nuclear greater than cytoplasmic), C>N (cytoplasmic greater than nuclear), and C (cytoplasmic). Transfection of NP-FL demonstrated a typical punctuate or dotted nuclear localization pattern as described in the literature (Fig. 5) [3,6]. The other 3 deletion constructs, obpNLS+NC, obpNLS+N-ter, and obpNLS+C-ter, that contained the obpNLS and devoid of both nNLS and cNLS were all localized exclusively and/or predominantly to the nucleus (Fig. 5). Though there was some cytoplasmic localization observed in < 10% of cells transfected with obpNLS+NC, it was also noted that all these cells had nuclear fluorescence greater than the cytoplasmic, suggesting a typical N>C localization pattern (Fig. 5).

#### b. bpNLS-1 and bpNLS-2 containing the wild-type Arg at aa 105 (R^105^) translocate to nucleus

**Figure 6:**
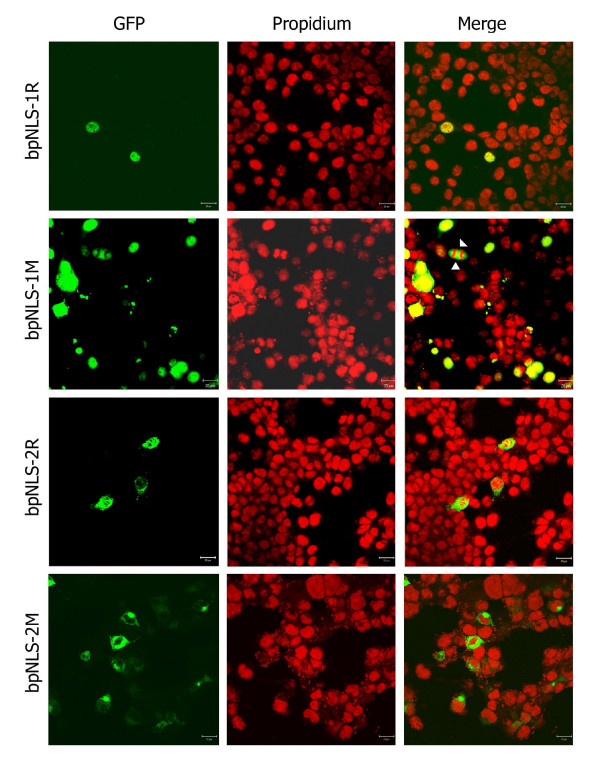
**Expression analysis of bpNLS-1 and bpNLS-2.** bpNLS-1R and bpNLS-2R (with R^105^) localizes predominantly to the nucleus. Note that the bpNLS-1M (with M^105^) exhibits a predominantly cytoplasmic localization pattern but also localizes to the nucleus as well. Arrows indicate a dividing cell with two daughter nuclei and the bpNLS-1M demonstrating an exclusive cytoplasmic localization. bpNLS-2M (with M^105^) localizes to the cytoplasm.

Since the obpNLS contained signals for two bpNLSs that were overlapping, we tested both the signals individually by separating them into bpNLS-1R and bpNLS-2R, such that the ^105^RR^106 ^of obpNLS was common to both these signals (Fig. 3). Transfection with these plasmid constructs resulted in the bpNLS-1R exhibiting a typical exclusive nuclear localization pattern (N), whereas the bpNLS-2R, though displaying a strong nuclear localization pattern however did also result in (though to a lesser extent) cytoplasmic localization as well (Fig. 6).

Once it was established that both bp-NLS-1R and bpNLS-2R (containing the wild-type Arg at position 105) were functional signals and were able to translocate efficiently to nucleus we attempted to evaluate the significance of Arg^105 ^in the nuclear localizing potential of both these signals. This was achieved by designing two plasmid constructs, with the Arg (R^105^) substituted with Met (M^105^), and were named bpNLS-1M and bpNLS-2M (Fig. 3). The bpNLS-1M-transfected cells demonstrated a C>N localization pattern and occasionally demonstrated an exclusive cytoplasmic localization in some cells (Fig. 6). Though the nuclear localization of bpNLS-1M was relatively less predominant than the cytoplasmic localization, this result was unexpected. This could be attributed to the fact that some proteins translocate efficiently to the nucleus even in absence of a functional NLS when the total basic residues content of the protein is greater than 20% [16,17]. This led us to analyze whether the basic residues within the bpNLS-1M aa sequence were contributing towards the nuclear localization. To our surprise, analyses of the bpNLS-1M aa sequence (constituting NP^41–106^) revealed that the total basic residue content within this region was exactly 20% and this as well might be the contributing factor in the nuclear localizing potential of bpNLS-1M. Similar findings have been reported previously wherein NP constructs containing aa 1–80 (a region rich in basic-residue content) localized exclusively to nucleus [7]. Analysis of the bpNLS-2M expression on the other hand revealed results as predicted and exhibited an exclusive cytoplasmic localization (Fig. 6).

#### c. NP-L constructs lacking the obpNLS localize predominantly to the cytoplasm

**Figure 7:**
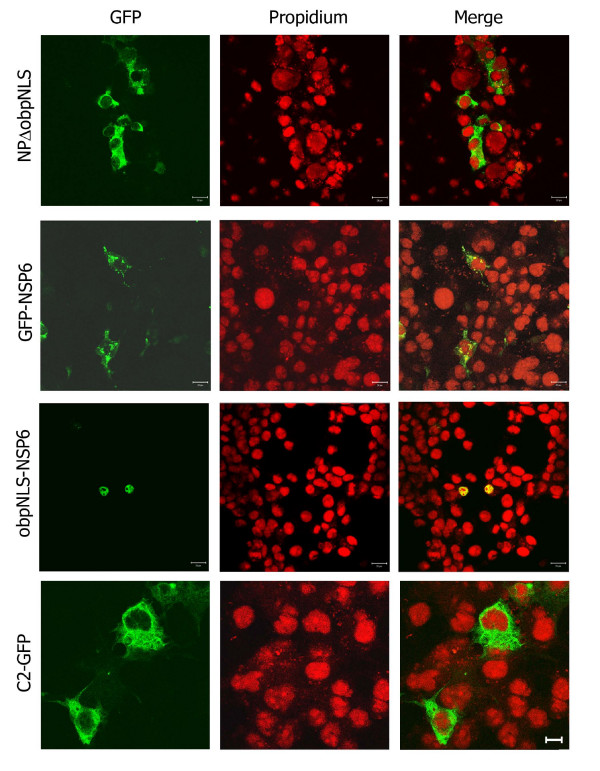
**Expression analysis of construct lacking obpNLS and construct with obpNLS in fusion with a known cytoplasmic protein.** NPΔ obpNLS, transfected in COS-7L cells, exhibits a characteristic cytoplasmic localization pattern. C2-GFP, used as a vector control, localizes to the cytoplasm. Rotavirus non structural protein 6 (NSP6) fused with GFP (GFP-NSP6) exhibits a typical punctate, cytoplasmic localization pattern. However, note that the obpNLS+NSP6 (obpNLS fused to the N-terminus of NSP6) exhibit an exclusive nuclear localization pattern. Bar represents 20 μm.

To further emphasize the significance of obpNLS in the nuclear localizing potential of NP a series of deletion constructs were made such that 3 different constructs, lacking all three NLSs (nNLS, cNLS and obpNLS), were achieved (Fig. 3). These 3 plasmids, NP^41–89^, NP^122–197 ^(data not shown) and NPΔ obpNLS, when transfected in to COS-7L cells revealed a cytoplasmic localization pattern as expected (Fig. 7). Interestingly, the NP^122–197 ^exhibited a cytoplasmic localization pattern (data not shown) similar to the bpNLS-2M (Fig. 6) with intensive accumulation in the cytoplasm as aggregates which appears more akin to golgi-like localization pattern.

#### d. obpNLS signal alone efficiently translocates a known cytoplasmic, non-influenza viral protein to the nucleus

In all the above experiments it was proven beyond doubt that the obpNLS was indeed a functional NLS with both the overlapping bpNLSs contributing equally to translocate the NP to nucleus. In order to ascertain that the obpNLS is a potent and functional NLS we tested the signal by fusing it to a known cytoplasmic protein, rotavirus NSP6 (Fig. 3) [21]. The rotavirus is a known cytoplasmic virus with exclusive cytoplasmic localization of all viral proteins. More specifically, the NSP6 exhibits a typical cytoplasmic, punctate localization pattern both in infected and transfected cells (Fig. 7) [21]. However, once the obpNLS was fused to NSP6, this known cytoplasmic protein localized exclusively to nucleus and demonstrated a typical nuclear localization pattern similar to that of the NP (Fig. 7).

Thus, the present study reports the presence of a novel third obpNLS in the influenza virus NP-L and unambiguously demonstrates that this signal is functional. It is noteworthy that the obpNLS of NP-L is present in 9 other influenza virus strains (out of a total of 500 NP sequences screened) suggesting that the Met to Arg change (M105R) does occur naturally and that this single aa change results in the conversion of the region between aa 90 to 121 to a 32-residue obpNLS (Table 2). It would be interesting to analyze whether the presence of the third NLS in NP in these strains impart any specific advantage to the virus or if there exists any difference in the nuclear staining pattern between strains with 2 and 3 NLSs in the NP. None of these 9 strains, containing obpNLS (Table 2), have been utilized in the previously published NP nuclear localization studies. However, it is to be noted that NP from type-B WSN33 strains (that contain only the nNLS and cNLS) were utilized extensively for cellular localization analysis and it was observed that the NP nuclear localization pattern was very similar in virus-infected as well as NP-transfected cells [3,6,8,14]. What is more pertinent is that the nuclear staining pattern observed with the NP-FL (containing 3 NLSs) in the present study is very similar to that observed with NP-FL (with 2 NLSs) in the previous studies [3,6,8,14]. Though this is an indirect evidence, it still provides a preliminary comparison of the staining pattern between these two types of NPs. Furthermore, of the 9 strains that exhibit the presence of an obpNLS, 6 are reassortant swine virus strains and the other 3 are WS/33 strains. Interestingly, two out of the 3 WS/33 strains, that contain the obpNLS exhibited distinct pathogenic features. One was a persistent, neurovirulent strain, while the second one was a temperature-sensitive variant (ts61). The precise role of obpNLS towards persistence or temperature-sensitivity of these strains is not known and needs to be investigated further [24].

**Table 2:** Details of the 9 Influenza A virus strains that contain a NP with obpNLS.

S. No	Accession number	Strain	Host	HN type	Features
1	Q1I2B5	A/WSN/1933 TS61	human	H1N1	Temperature-sensitive
2	Q1K9H2	A/WSN/1933	human	H1N1	N/A
3	Q9DLK6	A/WSN/1933	mice	H1N1	Persistent infection
4	Q5Q142	A/swine/Korea/S109/2004	swine	H9N2	N/A
5	Q5Q165	A/swine/Korea/S190/2004	swine	H9N2	N/A
6	Q5Q173	A/swine/Korea/S175/2004	swine	H1N1	N/A
7	Q5Q179	A/swine/Korea/S83/2004	swine	H9N2	N/A
8	Q5Q187	A/swine/Korea/S81/2004	swine	H9N2	N/A
9	Q5Q195	A/swine/Korea/S10/2004	swine	H1N1	N/A

HN type = hemagglutinin and neuraminidase classification type; N/A = data not available

## Conclusion

We report here a novel NLS in the NP of influenza A virus by bioinformatic analysis and present evidence that this signal is transport efficient. Future studies on the relevance of this signal towards viral replication or pathogenesis, especially by using a reverse-genetics system available for influenza virus, could provide valuable clues in viral biology. This report while not only establishes a new NLS in the influenza virus A strain, it also reinforces the idea that proper application of bioinformatics-coupled experimental analysis serves as a powerful tool in identifying new functional signals in proteins of interest [25].

## Availability and requirements

Institute for Medical Science:  

## Authors' contributions

KVKM identified the signal, conceived and designed experimental set up, performed experiments and drafted manuscript. CDA revised manuscript critically and approved final version. All authors read and approved the final manuscript.
